# Enabling comprehensive optogenetic studies of mouse hearts by simultaneous opto-electrical panoramic mapping and stimulation

**DOI:** 10.1038/s41467-021-26039-8

**Published:** 2021-10-04

**Authors:** Michael Rieger, Christian Dellenbach, Johannes vom Berg, Jane Beil-Wagner, Ange Maguy, Stephan Rohr

**Affiliations:** 1 Department of Physiology, University of Bern, Bühlplatz 5, Bern, Switzerland; 2 Institute of Laboratory Animal Science, University of Zürich, Wagistrasse 12, Schlieren, Switzerland

**Keywords:** Extracellular recording, Fluorescence imaging, Optogenetics, Cardiovascular biology

## Abstract

During the last decade, cardiac optogenetics has turned into an essential tool for investigating cardiac function in general and for assessing functional interactions between different myocardial cell types in particular. To advance exploitation of the unique research opportunities offered by this method, we develop a panoramic opto-electrical measurement and stimulation (POEMS) system for mouse hearts. The core of the experimental platform is composed of 294 optical fibers and 64 electrodes that form a cup which embraces the entire ventricular surface of mouse hearts and enables straightforward ‘drop&go’ experimentation. The flexible assignment of fibers and electrodes to recording or stimulation tasks permits a precise tailoring of experiments to the specific requirements of individual optogenetic constructs thereby avoiding spectral congestion. Validation experiments with hearts from transgenic animals expressing the optogenetic voltage reporters ASAP1 and ArcLight-Q239 demonstrate concordance of simultaneously recorded panoramic optical and electrical activation maps. The feasibility of single fiber optical stimulation is proven with hearts expressing the optogenetic voltage actuator ReaChR. Adaptation of the POEMS system to larger hearts and incorporation of additional sensors can be achieved by redesigning the system-core accordingly.

## Abstract (web summary)

Current cardiac mapping systems provide either electrical or optical readouts. Here the authors report a panoramic opto-electrical measurement and stimulation (POEMS) system which embraces the entire ventricular surface of mouse hearts, allowing flexible combinations of optical and electrical recording and stimulation modalities.

## Introduction

The measurement and modulation of membrane potentials of cardiac cells expressing optogenetic reporters and actuators of transmembrane voltage (*V*_m_) have turned into an established method in basic and translational cardiac electrophysiology in recent years^1,2^. The considerable potential of this method for making new discoveries in cardiac electrophysiology has been demonstrated by findings such as the observation of modulation of impulse conduction in the distal atrioventricular node by electrotonically coupled macrophages and the presence of electrical coupling of noncardiomyocytes to cardiomyocytes in the border zone of cryoinjuries^3,4^. Also, the feasibility of optical pacing of hearts or optical termination of arrhythmias with optogenetic actuators exerting a depolarizing effect on *V*_m_ of cardiomyocytes has been proven^5–7^. Full and efficient exploitation of the unique research opportunities offered by cardiac optogenetics for understanding heart function in health and disease demands an experimental method that combines panoramic optical and electrical imaging and stimulation of small rodent hearts into one system. While available recording systems provide either electrical or optical readouts and are designed primarily for larger hearts^8–15^, the panoramic optoelectrical measurement and stimulation (POEMS) system presented in this study integrates both modalities into a single experimental platform. Here, we show that the POEMS system enables straightforward high-content characterization of mouse ventricular electrophysiology in optically and electrically stimulated hearts from transgenic mouse models expressing the genetically engineered *V*_m_ indicators (GEVIs) ASAP1^16^ and ArcLight-Q239^17^ and the optogenetic *V*_m_ actuator ReaChR^18^ in cardiomyocytes.

## Results

### Components of the POEMS system

#### Heart container

**Fig. 1:**
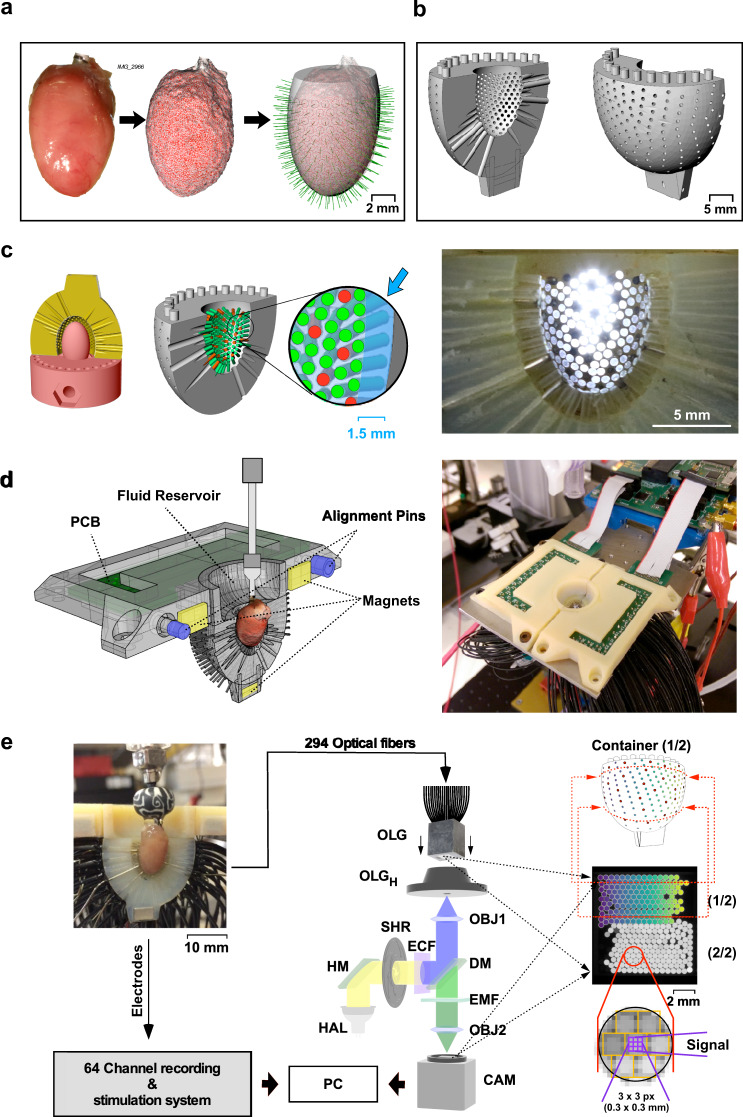
POEMS system components. **a** The heart container geometry was derived from adult mouse hearts (left) undergoing photogrammetry (center) with results being approximated by a tri-axial ellipsoid (right; prospective sensor locations indicated by green lines). **b** Design of one container half. **c** Left: for probe assembly, the container half (yellow) was temporarily fixed to a 3D-printed heart model (red) that aligned the probe tips during mounting to the prospective heart surface. Center: container with inserted probes (green: optical, red: electrical) with magnification depicting the fluid layer beneath the probes (blue arrow). Right: image of the fully assembled container half (bright discs: illuminated optical fibers, black discs: electrodes). **d** Left: visualization of one-half of the platform assembly with printed circuit board (PCB) holder, fluid reservoir, and organ container. Right: fully assembled POEMS system. **e** System overview. Left: the 64 electrical probes are connected to a dedicated recording/stimulation system. Center: the faceplate of the combined 294 optical light guides (OLG) is fixed by a holder (OLG_H_) to the image plane of a tandem-lens epifluorescence macroscope (HAL halogen lamp, HM hot mirror, SHR shutter, ECF excitation filter, DM dichroic mirror, EMF emission filter, OBJ1,2 macroscope objectives, CAM camera). Right, center: view of the OLG faceplate with gray and colored fiber stacks belonging to one container half each. Horizontally aligned fibers follow coronal sections of the container (upper schematic). The enlargement shown at the bottom depicts the assignment of sensor pixels to individual fibers and illustrates an example of a 3 × 3 pixel array capturing the light from a single optical fiber.

The core of the POEMS system is formed by a cup-shaped container whose dimensions were derived from 3D reconstructed hearts of adult mice (Fig. 1a). The positioning of optical fibers and electrodes on the heart surface was defined by a circle-packing algorithm that generated 358 evenly spaced prospective measurement and stimulation sites at a pitch of 0.7 mm. Based on the 3D organ and detector site information, the final container was designed with the inner surface being separated by 1.5 mm from the prospective heart surface (Supplementary Fig. 1). The container was split in half (Fig. 1b) and, after 3D printing, temporarily attached to a 3D-printed heart model that served as an insertion stop during probe mounting (Fig. 1c). This stop caused the ends of the inserted optical fibers (294 polymethyl methacrylate fibers, diameter: 500 µm) and electrodes (64 PTFE coated silver wires, diameter: 380 µm core, 500 µm with insulation) to form the actual heart interface with the space beneath permitting epicardial solution exchange during experiments. The system was completed by mounting each container half to a fluid reservoir that was part of the platform holding the printed circuit boards (PCBs) for the electrical connections (Fig. 1d).

#### Electrical subsystem

Electrode signals were routed to a custom-built electrical recording and stimulation system (Fig. 1d, e) that provided 64 analog input/output channels and signal processing functions. Unipolar electrograms were recorded at 10 kHz with the aortic cannula acting as a reference electrode. For stimulation purposes, the system delivered user-defined source- and sink currents to any of the 64 electrodes. A custom-developed field-programmable gate array (FPGA) system linked the components of the electrical subsystem to a PC where dedicated software served to control the recording of electrograms and the stimulation of the preparations. Also, the software permitted visualization of selected electrograms in real-time.

#### Optical subsystem

The distal ends of the optical light guides were assembled into 20 planar sheets containing sequentially ordered fibers that followed coronal sections of the container (Fig. 1e, right). The planar sheets were bonded and cut flat with a diamond mill. The resulting faceplate was mounted in the image plane of a custom-made epifluorescence macroscope (Fig. 1e, center). Excitation light was provided by a tungsten halogen lamp and filtered according to the specifications of the voltage indicators used. Emitted fluorescence was recorded by a high-speed CMOS camera. Optical and electrical measurements and stimulations were synchronized by TTL signals.

#### Analysis software

Data processing and visualization were performed offline with custom-developed software. Single-pixel optical data were automatically assigned to individual fibers by using an extraction mask that matched the pixels to individual fibers within the faceplate (Fig. 1e). Subsequent data processing included filtering, action potential (AP) detection, and determination of AP characteristics, including fractional fluorescence changes (d*F*/*F*), maximal upstroke velocities (d*V*/d*t*_max_), and durations (APD). For unipolar electrograms, the software extracted the amplitude and the duration of the negative deflection which indicated local electrical activation. Panoramic activation maps were constructed based on local activation times (LATs). They were derived either from the time of the peak of the Savitzky–Golay derivative (eLAT_SG_, oLAT_SG_) of the signals^19,20^ or the time when optical AP upstrokes reached 50% of their full amplitude (oLAT_50%_). Optical and electrical LATs were compared by assessing the difference between eLAT_SG_ of a given electrode and the mean of oLAT_50%_ or oLAT_SG_ of all six optical fibers surrounding that electrode.

Details regarding the hardware, data-analysis procedures, experimental protocols, and the generation of transgenic ArcLight-Q239^17^ and ASAP1^16^ mouse models are provided in “Methods”.

### Simultaneous optoelectrical determination of ventricular activation of a di-8-ANEPPS stained C57BL/6J mouse heart in sinus rhythm

**Fig. 2:**
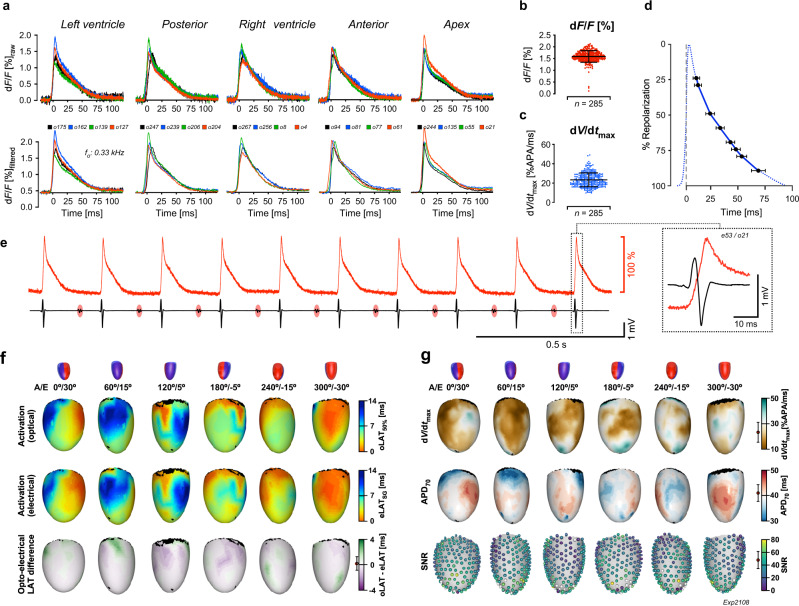
Panoramic optoelectrical mapping of a di-8-ANEPPS stained C57BL/6J mouse heart in sinus rhythm. **a** Raw and filtered optical action potential (oAPs) as recorded by a selection of individual optical fibers in contact with different regions of the heart. **b** Fractional fluorescence changes (d*F*/*F*) of oAPs. **c** Maximal upstroke velocities (d*V*/d*t*_max_) of oAPs. **d** Durations of oAPs measured at different levels of repolarization (mean ± SD; *n* = 285). **e** Sequence of sinus activations measured simultaneously by a single optical fiber (above) and an adjacent electrode (below). Large amplitude electrograms indicating local ventricular activation are preceded by small signals corresponding to atrial activation (red ovals). The last activation is shown in expanded form on the right. **f** Schematic of heart orientation (A: angle, E: elevation, red: left ventricle, blue: right ventricle) with corresponding 3D maps of optically (top) and electrically (center) determined ventricular activation patterns. Bottom: Difference map of optical and electrical determinations of local activation times (oLAT_50%_ − eLAT_SG_) with mean ± SD shown on the left of the color bar (*n* = 64). **g** 3D maps of the spatial distribution of d*V*/d*t*_max_ (top), action potential duration at 70% repolarization (APD_70_; center) and signal-to-noise ratios (SNRs) of oAPs recorded during a single activation with spheres indicating the recording sites. Parameter statistics (mean ± SD) are shown on the left of the color bars (*n* = 285 each). “*n*“ refers to the number of signals produced by the recording sites during a single activation of the heart. Error bars refer to ±SD. Source data are provided as a Source Data file.

Raw and low-pass filtered action potentials recorded optically at 10 kHz from all main regions of a di-8-ANEPPS stained mouse heart in sinus rhythm are shown in Fig. 2a. Optical action potentials (oAPs; *n* = 285) recorded by the fibers during a single activation of the heart exhibited a mean d*F*/*F* of 1.59 ± 0.25%, maximal upstroke velocities (d*V*/d*t*_max_) of 23.12 ± 7.13 %APA/ms and a duration at 70% repolarization (APD_70_) of 42.19 ± 4.05 ms (Fig. 2b–d). A sequence of ten sinus activations recorded simultaneously by a single optical fiber and an adjacent electrode is shown in Fig. 2e. The presence of sinus rhythm at a frequency ~5 Hz is confirmed by low-amplitude far-field electrograms reflecting atrial activation that regularly precede the larger deflections associated with local ventricular excitation. The expanded view of an oAP upstroke overlaid on the unipolar electrogram of an adjacent electrode demonstrates concurrence of the oAP upstroke and the electrogram downstroke. Figure 2f depicts panoramic views of ventricular activation that are simultaneously recorded by the optical and electrical subsystems. As shown by both maps, initial breakthroughs of activation occurred both on the lateral left ventricular wall and in the basal–posterior region of the right ventricle. The latest activations were observed on the lateral right ventricular wall (for animation cf. Supplementary Movie 1 and Supplementary Movie 2). Overall ventricular activation took 12.1 ms (optical) and 12.8 ms (electrical). The concordance between optical and electrical activation maps is illustrated by sub-millisecond differences between oLATs and eLATs (0.21 ± 1.07 ms; *n* = 64 electrode sites) which demonstrates the capability of the POEMS system to capture ventricular activation with high fidelity irrespective of the recording modality used. Additional parameters characterizing ventricular activation and overall signal quality are depicted in Fig. 2g. While the spatial pattern of d*V*/d*t*_max_ showed no noticeable dependence on the ventricular activation sequence, APD_70_ tended to be the longest at the site of initial left ventricular excitation. Signal-to-noise ratios (SNRs) of optical signals amounted to 48.8 ± 13.7 (unfiltered data; *n* = 285 oAPs) with the majority of fibers (>98%) reporting oAPs with an SNR between 20 and 80 (Supplementary Fig. 2). Mean SNRs of right ventricular oAPs (*n* = 109) were slightly lower (−6.9%) than those of left ventricular oAPs (*n* = 176; *P* = 0.028).

### POEMS mapping of electrically stimulated hearts expressing the optogenetic voltage reporters ArcLight-Q239 and ASAP1

**Fig. 3:**
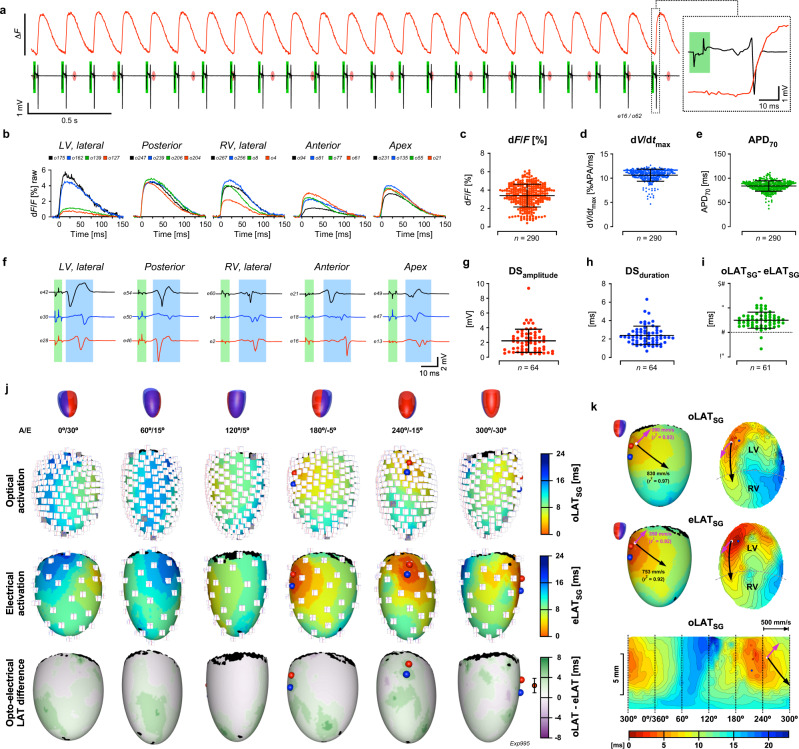
Panoramic optoelectrical recordings from an electrically stimulated Myh6-Cre^Tg^/ArcLight-Q239^lox/wt^ heart. **a** Optical trace (top) and electrical trace (bottom; red ovals: atrial activity; green rectangle: stimulation artifact) as recorded simultaneously by a single optical fiber and by an adjacent electrode during epicardial stimulation at  6 Hz. The last activation is shown in expanded form on the right. **b** Unfiltered optical action potentials (oAPs) recorded by a selection of individual fibers that image different regions of the heart (LV: left ventricle; RV right ventricle). **c** Fractional fluorescence changes (d*F*/*F*) of oAPs. **d** Distribution of maximal upstroke velocities (d*V*/d*t*_max_) of oAPs. **e** Durations of oAPs measured at 70% repolarization (APD_70_). **f** Electrograms of different regions of the heart (green bands: stimulation artifact; blue bands: local electrogram). **g** Distribution of downstroke (DS) amplitudes and **h** downstroke durations of all electrograms recorded during a single ventricular activation. **i** Distribution of local differences between optically and electrically determined local activation times (oLAT_SG_ − eLAT_SG_). **j** Schematic of heart orientation (A: angle; E: elevation; red: left ventricle; blue: right ventricle) with corresponding 3D maps of optically determined activation (top; with miniaturized signals at the recording sites), electrically determined activation (center), and optoelectrical LAT differences (bottom) with mean ± SD values shown on the left of the color bar (*n* = 61). The location of the stimulation electrodes is indicated by a red and blue sphere, respectively. **k** 3D map and central projection of optically (top: oLAT_SG_) and electrically (center: eLAT_SG_) determined ventricular activation sequences. Bottom: Mercator projection of the optically determined activation sequence. White disc: site of initial activation; black arrow: direction of fastest activation; red arrow: propagation perpendicular to the black arrow (arrow lengths are scaled to the conduction velocities). “*n*” refers to the number of signals produced by the recording sites during a single activation of the heart. Error bars refer to ±SD. Source data are provided as a Source Data file.

Hearts of Myh6-Cre^Tg^/ArcLight-Q239^lox/wt^ animals were stimulated on the free left ventricular wall while undergoing panoramic optoelectrical mapping. Figure 3a depicts a sequence of stimulated action potentials that was simultaneously recorded by a single optical fiber at 1 kHz and an adjacent electrode. Sinus activity (~4.1 Hz) marked by red oval discs in the electrogram was overdriven by stimulating the ventricles at 6 Hz (green rectangles). The expanded view of the optical and electrical signal on the right reveals that the oAP upstroke was delayed in respect to the fast negative deflection of the electrogram. This is explained by the relatively slow kinetics of ArcLight-Q239 that also blunted the overshoot of the AP (Fig. 3b). Compared to di-8-ANEPPS, ArcLight-Q239 exhibited a higher d*F*/*F* of 3.41 ± 1.24% (Fig. 3c; *n* = 290) and an improved SNR of unfiltered signals of 78.5 ± 34.0 (*n* = 290). By contrast, d*V*/d*t*_max_ was reduced to 10.62 ± 1.25 %APA/ms and APD_70_ prolonged to 83.90 ± 10.73 ms (Fig. 3d, e; *n* = 290). Ventricular electrograms were preceded by the stimulation artifact (Fig. 3f, green bands). Stimulation artifacts were minimized by transiently increasing the AC-coupling frequency of the amplifiers during stimulation and by adjusting the negative and positive components of the biphasic current pulse. These procedures permitted eLAT determinations also in the vicinity of the stimulation electrodes (e.g., trace e_42_ in Fig. 3f was recorded at a distance of 1.3 mm from to stimulation site). Electrograms were characterized by a mean amplitude of 2.25 ± 1.60 mV and a downstroke duration of 2.34 ± 1.03 ms (Fig. 3g, h; *n* = 64). As illustrated by the 3D activation maps shown in Fig. 3j, optically and electrically determined activation patterns were qualitatively similar (for animation cf. Supplementary Movie 3). However, the slow kinetics of ArcLight-Q239 caused oLAT_50%_ to be consistently delayed in respect to eLAT_SG_ by 2.42 ± 1.67 ms (Fig. 3i and bottom panel of Fig. 3j; *n* = 61). Stimulated ventricular activation lasted for 23.1 ms (optical) and 19.5 ms (electrical). The 3D, central-, and Mercator projections of ventricular activation depicted in Fig. 3k show that excitation elicited at the location marked with a white sphere spread preferentially in latero-apical direction with maximal conduction velocities (*θ*) measured optically (*θ*_o_) of 830 mm/s and electrically (*θ*_e_) of 753 mm/s. Conduction velocities determined in perpendicular direction reached 390 mm/s (*θ*_o_) and 350 mm/s (*θ*_e_) resulting in anisotropy ratios of 2.13 and 2.15 for optical and electrical recordings, respectively.

**Fig. 4:**
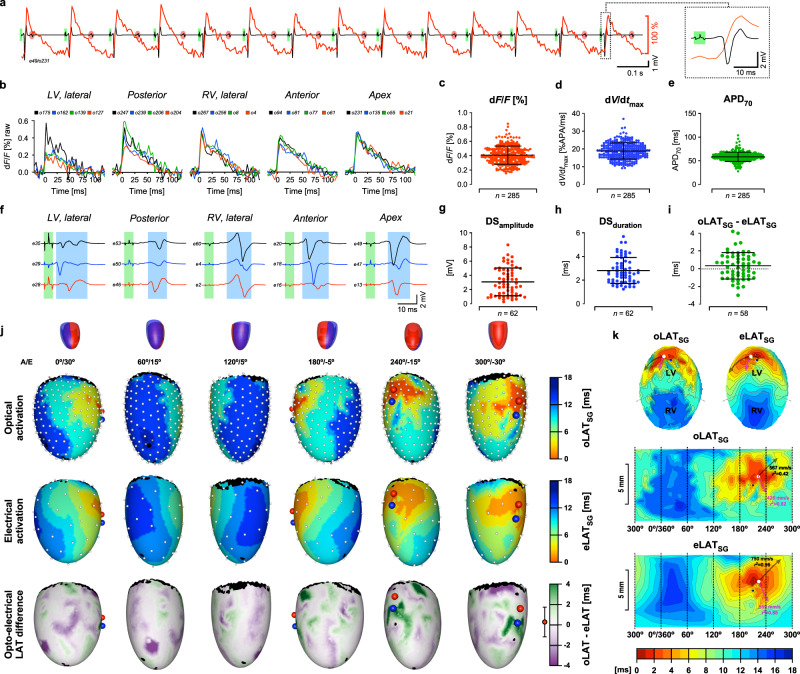
Panoramic optoelectrical recordings of stimulated activation of a Myh6-Cre^Tg^/ASAP1^lox/wt^ heart. **a** Single fiber optical signal overlaid on the electrogram of an adjacent electrode (red oval: atrial activity; green rectangle: stimulation artifact). The last activation is shown in expanded form on the right. **b** Unfiltered optical action potentials (oAPs) recorded by a selection of optical fibers in contact with different regions of the heart. **c** Fractional fluorescence change (d*F*/*F*) of oAPs. **d** Maximal upstroke velocities (d*V*/d*t*_max_) of oAPs. **e** Duration of oAPs determined at 70% of repolarization (APD_70_). **f** Electrograms recorded from the regions of the heart indicated (green bands: stimulation artifact; blue bands: local electrogram). **g** Distribution of downstroke (DS) amplitudes and **h** downstroke durations of the electrograms recorded during a single ventricular activation. **i** Distribution of local differences between oLAT_SG_ and eLAT_SG_. **j** Schematic of heart orientation (A: angle; E: elevation; red: left ventricle; blue: right ventricle) with corresponding 3D maps of the optically determined activation sequence (top), electrical activation (center), and the differences between optically and electrically determined local activation times (oLAT − eLAT) (bottom; mean ± SD shown on the left of the color bar; *n* = 58). The location of the stimulation electrode pair is indicated by a red and blue sphere, respectively. **k** Central- and Mercator projections of ventricular activation determined optically (oLAT_SG_) and electrically (eLAT_SG_). White discs: site of initial activation; black arrow: direction of fastest activation; red arrow: propagation perpendicular to the black arrow (arrow lengths are scaled to the conduction velocities). “*n*” refers to the number of signals produced by the recording sites during a single activation of the heart. Error bars refer to ±SD. Source data are provided as a Source Data file.

An identical experiment with a heart from a Myh6-Cre^Tg^/ASAP1^lox/wt^ animal is shown in Fig. 4. As illustrated by the single-fiber optical recording overlaid on the electrogram of a nearby electrode (Fig. 4a), sinus activity (5.3 Hz to 6.7 Hz, irregular; red ovals) was overpaced at 7 Hz (green rectangles). Optical APs recorded from different regions of the heart showed typical shapes (Fig. 4b) but were small (d*F*/*F* 0.41 ± 0.13%, *n* = 285; Fig. 4c) which required the use of increased integration times (frame rate: 250 Hz) and led to a moderate SNR of the unfiltered signals of 16.6 ± 7.3. Owing to the faster kinetics compared to ArcLight-Q239, ASAP1 reported higher maximal upstroke velocities (18.88 ± 4.48 %APA/ms; Fig. 4d) and a shorter APD_70_ (58.51 ± 9.55 ms, *n* = 285; Fig. 4e). Simultaneously recorded electrograms (Fig. 4f) exhibited downstroke amplitudes (3.09 ± 1.98 mV, *n* = 62; Fig. 4g) and durations (2.79 ± 1.11 ms; Fig. 4h) similar to those recorded from ArcLight-Q239 expressing hearts. From the stimulation site, excitation spread uniformly across the ventricles with electrical and optical activation patterns being highly similar as demonstrated by the small oLAT_SG_-eLAT_SG_ difference of 0.35 ± 1.50 ms (*n* = 58; Fig. 4i, j; for animation cf. Supplementary Movie 3). The central- and Mercator projections shown in Fig. 4k illustrate that maximal conduction velocities determined by either measurement modality were comparable (*θ*_o_: 567 mm/s; *θ*_e_: 750 mm/s) as were anisotropy ratios (optical: 1.33; electrical: 1.34) and overall activation times (optical: 16.0 ms; electrical: 16.7 ms).

### Single-fiber optical stimulation of a heart expressing the optogenetic membrane voltage actuator ReaChR

**Fig. 5:**
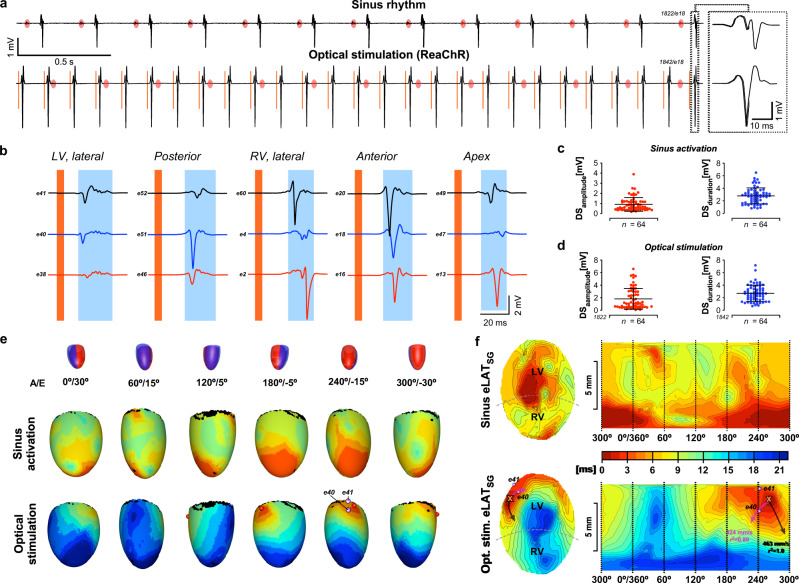
Single fiber optical stimulation of a Myh6-Cre^Tg^/ReaChR^lox/wt^ heart. **a** Local unipolar electrograms during sinus rhythm (upper trace; red ovals: atrial activations) and during optical stimulation (lower trace; identical recording site; orange lines: optical stimulation). The last electrogram is shown in expanded form on the right. **b** Selection of electrograms recorded at different sites (LV: left ventricle; RV: right ventricle) after optical stimulation (stimulation: orange band; local electrograms: blue bands). **c** Electrogram downstroke (DS) amplitudes and durations recorded during sinus rhythm and, **d** during optical stimulation. **e** Schematic drawings of heart orientation (A: angle; E: elevation; red: left ventricle; blue: right ventricle) with corresponding 3D maps of sinus driven ventricular activation (top) and optically stimulated activation (bottom; red sphere: site of optical stimulation; violet spheres: recording electrodes close to the stimulation site with signals shown in panel b). **f** Central- and Mercator projections of electrically determined ventricular activation (eLAT_SG_) during sinus rhythm (top) and following optical stimulation (bottom; orange cross: optical stimulation site; violet spheres: electrodes; black arrow: direction of fastest activation; red arrow: propagation in perpendicular direction; arrow lengths are scaled to conduction velocities). “*n*” refers to the number of signals produced by the recording sites during a single activation of the heart. Error bars refer to ±SD. Source data are provided as a Source Data file.

Optical stimulation capabilities of the POEMS system were validated with hearts of Myh6-Cre^Tg^/ReaChRr^lox/wt^ mice. After control recordings in presence of sinus rhythm (Fig. 5a, upper panel; atrial activity indicated by red ovals), the heart was optically stimulated using a single fiber delivering 5 ms long pulses of excitatory light (590 nm) with a total power of 1.7 mW (8.8 mW/mm^2^). Successful optical overdrive pacing at 7 Hz is demonstrated by constant intervals between the optical pulse (orange line) and the field potential reported by a single electrode (Fig. 5a, lower trace). The expanded view of electrograms acquired by the same electrode during sinus rhythm and during optical stimulation shows a change of shape during optical stimulation which is likely explained by the concomitant change in activation direction. The comparison of electrogram parameters determined during optical stimulation and sinus rhythm (Fig. 5c, d) shows similar downstroke times (2.79 ± 1.28 ms vs. 2.74 ± 1.32 ms, *n* = 64), whereas downstroke amplitudes were substantially larger during optical stimulation (1.84 ± 1.64 mV vs. 0.91 ± 0.68 mV, *n* = 64). The 3D activation maps shown in Fig. 5e demonstrate initiation of activation at the optical stimulation site from where it invaded the ventricles with the basal regions of the lateral right ventricle being excited last (for animation cf. Supplementary Movie 4). Overall ventricular activation took 11.6 ms (sinus rhythm) and 20.9 ms (optical stimulation). As illustrated by the central- and Mercator projections (Fig. 5f), maximal *θ*_e_ during optical stimulation amounted to 463 mm/s with *θ* perpendicular to this vector amounting to 324 mm/s (anisotropy ratio: 1.43).

### Concordance of optical and electrical LATs

**Fig. 6:**
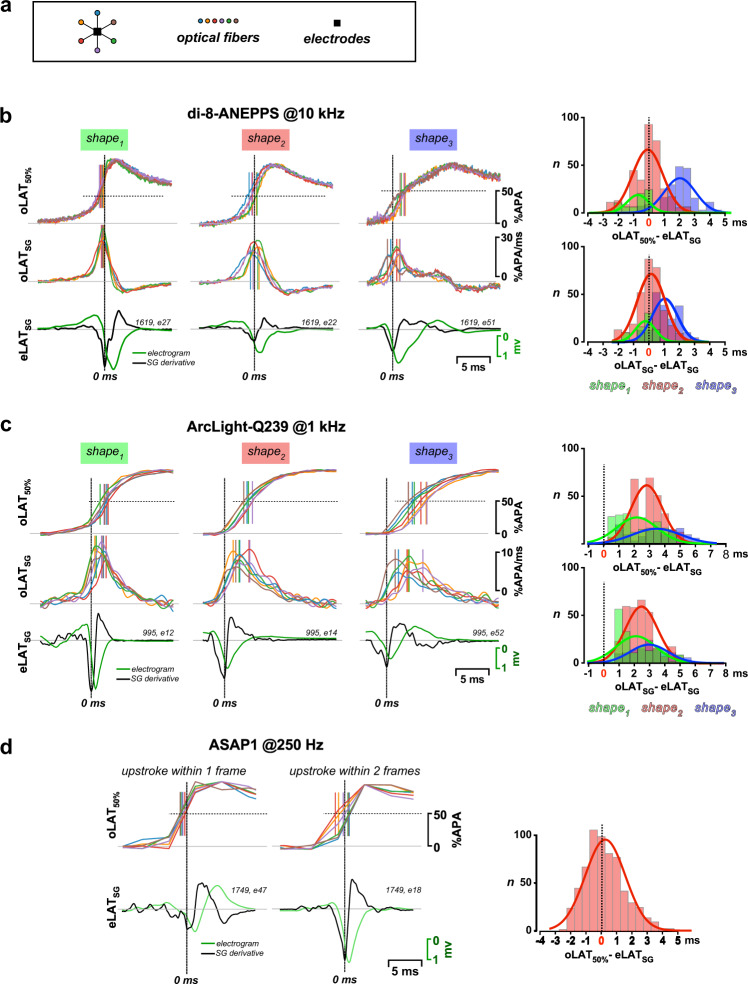
Concordance of optical and electrical LAT determinations. **a** Schematic drawing of the sensor layout used for the determination of optoelectrical differences in local activation time (LAT). **b** Optoelectrical LAT differences for a heart stained with di-8-ANEPPS. Left side, top: example traces of prototypical optical action potential (oAP) upstrokes classified into shapes 1–3 with vertical lines indicating oLAT_50%_ of each signal. Left side, center: same for SG (Savitzky-Golay) derivatives of the oAPs. Left side, bottom: electrogram of the central electrode (green) with overlaid SG derivative (black) and eLAT_SG_ set to zero. Right side: histograms of optoelectrical LAT differences for oLAT_50%_ (top) and oLAT_SG_ (bottom) with data being grouped according to shapes_1-3_. **c** Same as (**b**) for an ArcLight-Q239 expressing heart. **d** Same as (**b**) for an ASAP1 expressing heart. The low sampling frequency required for ASAP1 precluded meaningful shape classifications and oLAT_SG_ determinations. Source data are provided as a Source Data file.

Ideally, LATs derived from optical and electrical measurements are exactly matching. We tested the respective performance of the POEMS system by conducting simultaneous dual-mode recordings in hearts stimulated on the free left ventricular wall at 5 Hz. Optical and electrical LATs of ten sequential activations were compared by assessing the difference between eLAT_SG_ reported by each electrode and the mean of oLAT_50%_ or oLAT_SG_ of all six optical fibers surrounding that particular electrode (Fig. 6a). The results obtained with a di-8-ANEPPS stained heart are shown in Fig. 6b. Upstrokes of oAPs could be classified into three shapes that differed in respect to their optoelectrical concordance. Upstrokes showing a distinct foot potential followed by a rapidly rising phase (shape_1_; *n* = 70) were advanced relative to eLAT_SG_ by −0.69 ± 0.71 ms (oLAT_50%_) and −0.23 ± 0.67 ms (oLAT_SG_). LATs of upstrokes exhibiting a uniform rise (shape_2_; *n* = 332) closely coincided with eLAT_SG_ (oLAT_50%_: −0.07 ± 0.99 ms; oLAT_SG_: 0.14 ± 0.87 ms). Shape_3_ upstrokes (*n* = 187) showed two phases consisting of an initially fast-rising component followed by a second slower upstroke to the peak. These signals were found exclusively over the right ventricle and likely reflect asynchronous activation of the septum and the free right ventricular wall. Because of their particular shape, oLAT_50%_ of shape_3_ signals tended to be delayed relative to eLAT_SG_ (2.03 ± 1.02 ms) while oLAT_SG_, by capturing the initial fast phase, trailed eLAT_SG_ to a lesser extent (1.04 ± 0.80 ms; *n* = 187). The latter finding is reflected in the corresponding histograms by the smaller dispersion of the peaks for oLAT_SG_ determinations. Similar classes of upstrokes were also observed in the hearts of Myh6-Cre^Tg^/ArcLight-Q239^lox/wt^ mice (Fig. 6c). They were less distinct because the moderate on-kinetics of ArcLight-Q239 led to a smoothing of the multiphasic upstrokes of shape_3_ signals. The moderate kinetics also led to trailing oLATs in respect to the eLATs which, even when using oLAT_SG_, reached up to 3 ms for shape_3_ upstrokes. By contrast to ArcLight-Q239, the faster responding ASAP1 showed improved concordance between oLAT_50%_ and eLAT_SG_ (0.25 ± 1.31 ms; *n* = 628; Fig. 6d). Shape classifications and oLAT_SG_ determinations could not be performed because the long integration times (4 ms) needed for the small ASAP1 signals caused oAP upstrokes to extend over 1–2 sampling periods only thereby masking multiphasic upstrokes and excluding a meaningful determination of the time of d*V*/d*t*_max_. Overall, the results demonstrate that the electrical and optical recording modalities of the POEMS system yield virtually identical LATs for uniformly rising APs detected with fast voltage indicators. The results furthermore indicate that optoelectrical correspondence can be compromised over the right ventricle because of optical crosstalk between ventricular and septal signals.

## Discussion

The POEMS system constitutes an experimental platform that enables efficient and comprehensive ‘drop&go’ electrophysiological studies of wild-type and genetically modified mouse hearts. The system combines spatiotemporally controlled focal or global electrical and/or optical stimulation with simultaneous panoramic electrical mapping and/or optical imaging of any parameter of interest for which optical indicators are available.

Compared to conventional camera-based panoramic imaging systems^8–13^, fiber-optic contact mapping as implemented in the POEMS system offers distinct advantages: (i) organ positioning is straightforward and focusing is not required; (ii) collected data are inherently three-dimensional thereby eliminating the need for transforming 2D sensor data into 3D coordinates of the heart; (iii) illumination and signal pickup are uniform; (iv) light delivery and collection can be optimized by using high numerical objectives with short focal distances that cannot be used in multi-camera setups due to steric hindrance; (v) fiber-optic imaging can be combined with other sensors of interest; (vi) precise 3D correlation of recording sites and heart surface is feasible by inducing small burn marks using a subset of electrodes at the end of the experiment; (vii) the wet organ environment can be entirely separated from delicate recording systems; (viii) the system can be precisely matched to individual hearts by producing differently sized containers. Because containers can be exchanged in less than a minute, the appropriate size can be selected and mounted after determining the size of the isolated Langendorff heart. Disadvantages of the fiber-optic approach include a reduction of emitted fluorescence by ~1/3 compared to direct epifluorescence imaging due to obligatory losses associated with light guides and a suboptimal finish of the fiber ends. Also, if several optogenetic constructs are simultaneously expressed, the problem of wavelength congestion persists. Finally, the diameters of optical fibers and electrode wires chosen limit the spatial resolution of the POEMS system presented to 620 µm (optical) and 1180 µm (electrical). Improving the resolution to 100 µm by using thinner electrode wires as well as thinner optical fibers that match the pixel size of the camera (100 × 100 µm) is feasible as demonstrated by single-pixel raw signals that exhibited SNRs >20 (di-8-ANEPPS; Supplementary Fig. 3). Also, simultaneous optoelectrical mapping of ventricles and atria may be implemented by re-designing the heart container accordingly.

Validation of the POEMS system using simultaneous optoelectrical recording showed that panoramic ventricular activation maps can be obtained with any of the *V*_m_ indicators used. Failure rates for optical and electrical recording sites ranged between 0 and 4%. Full interchangeability of optical and electrical activation maps was demonstrated by oLATs differing from eLATs by less than a millisecond when using fast indicators^21,22^. Optical activation maps based on the slower responding GEVI ArcLight-Q239 matched simultaneously acquired electrogram maps qualitatively but were delayed as a whole by ~2–3 ms. Di-8-ANEPPS and ArcLight-Q239 recordings revealed that the precision of oLAT determinations over the thin right ventricle can be compromised by asynchronous activation of the underlying septum. If present, oLAT_SG_ determinations are to be preferred over oLAT_50%_ determinations because they are less sensitive to septal interference. Overall ventricular activation times of hearts in sinus rhythm measured either optically or electrically were within the range of QRS values reported before^23–25^ as was maximal *θ* following epicardial stimulation^26–28^ and anisotropy ratios^25,26^. Optically determined AP upstroke velocities were largest for di-8-ANEPPS measurements and matched those determined before by camera-based direct imaging of RH-237 stained mouse hearts^29^. Compared to published data, APDs tended to be slightly prolonged which is a known side-effect of blebbistatin that was routinely used in the experiments^30^ (Supplementary Fig. 4). Finally, amplitudes, downstroke durations, and maximal negative slopes of local electrograms recorded by the POEMS system were in the range of values reported for unipolar recordings before^31–33^. Beyond optoelectrical mapping and electrical stimulation, the POEMS system supported single fiber optical stimulation of hearts expressing ReaChR in cardiomyocytes. Power densities required for stable pacing were in the range of values reported before for optically paced hearts expressing channelrhodopsin-2^5,34^.

Compared to the GEVI VSFP2.3 used for assessing cardiac electrical activity in genetically modified animals before^28^, the transgene models generated in the context of this study produced either larger signals in response to an AP (ArcLight-Q239) or signals that reproduced the AP shape with higher fidelity (ASAP1). Similar to VSFP2.3, the disadvantage of ArcLight-Q239 concerns its slow kinetics that led to a suppression of AP overshoots and an artifactual AP prolongation based on a slower response to hyperpolarization than to depolarization^35^. With response kinetics being over an order of magnitude faster than ArcLight-Q239, ASAP1 produced oAP shapes comparable to those recorded in hearts stained with the ultrafast voltage-sensitive dye di-8-ANEPPS. The small nonlinearities of the Δ*F*–Δ*V*_m_ relationship reported for both optogenetic voltage indicators may have affected AP shape as well^12,17^. However, compared to the distortions of AP shape related to slow indicator kinetics, these effects were likely minimal.

The observation that fractional fluorescence changes (d*F*/*F*) per AP were eight times smaller for ASAP1 than for ArcLight-Q239 was unexpected because the two GEVIs have been reported before to share similar sensitivities when expressed in HEK cells^35^. To gain insights into this discrepancy, we analyzed the relative contributions of tissue autofluorescence, blebbistatin fluorescence, and GEVI fluorescence to the total resting fluorescence used for d*F*/*F* calculations. For this purpose, we determined tissue autofluorescence and blebbistatin fluorescence in hearts from wild-type mice (*n* = 3) and subtracted these values from the resting fluorescence measured in ArcLight-Q239 (*n* = 3) and ASAP1 (*n* = 5) expressing hearts to infer GEVI-specific fluorescence. Using this approach, we found that, on average, ArcLight-Q239 based fluorescence (47.9%) matched the sum of tissue (44.0%) and blebbistatin (8.1%) dependent fluorescence. By comparison, ASAP1 exhibited a reduced fluorescence of 11.8%. Under the assumption that the two GFP-based GEVIs exhibit similar excitation and emission characteristics, this suggests that ASAP1 was less efficiently expressed which is consistent with the moderate SNRs observed. The data furthermore demonstrate that tissue autofluorescence and blebbistatin acting as inevitable contributors to overall fluorescence in whole-heart measurements cause a substantial underestimation of true d*F*/*F*s by a factor of 2.1 for ArcLight-Q239 and 5.4 for ASAP1. After correction for this distortion, d*F*/*F* per action potential increases to 2.2% (ASAP1) and 7.1% (ArcLight-Q239) thereby reducing the gap to the 1:1 relation reported previously for transfected HEK cells where autofluorescence is minimal and blebbistatin is absent. Further studies are needed to understand why expression levels of ASAP1 seem to be lower than those of ArcLight-Q239. In summary, whereas ArcLight-Q239 excels in terms of signal amplitude, ASAP1, despite displaying lower SNRs, is preferable if oAP upstrokes, APDs and concordance between oLATs and eLATs are of interest.

In conclusion, the POEMS system enables straightforward panoramic optical and/or electrical imaging of the ventricles of small rodent hearts. Because recording sites are readily commutable to electrical or optical stimulation sites, the problem of ‘wavelength congestion’ associated with optogenetic constructs^36^ can be avoided by selecting appropriate combinations of electrical and optical stimulation and recording modalities. Besides being suited to validate optogenetic sensors and actuators with respect to co-localized electrical measurements, the POEMS system likely permits the recording of signals from optical reporters different from GEVIs like genetically engineered calcium indicators^37^ and can be used in conjunction with optogenetic actuators controlling, e.g., cell signaling^38^. Further conceivable applications include investigations of the dependence of arrhythmogenic slow conduction on optically measured Ca^2+^ inward currents^39^ and the elucidation of consequences of heterocellular electrotonic crosstalk between cardiomyocytes and noncardiomyocytes for cardiac activation and the precipitation of cardiac arrhythmias. Finally, the conceptual approach offers future flexibility because an increase of the spatial resolution, the inclusion of additional types of sensors, and the adaptation to larger hearts can be readily accomplished by redesigning the 3D-printed heart container only while keeping all other system components unchanged.

## Methods

### Heart container design

The POEMS system is based on a heart container that was dimensioned according to 3D reconstructed hearts of adult mice obtained by photogrammetry (C57BL/6J; 3–8 months; 2 male, 1 female; cf. Fig. 1a). Based on the mean heights of the ventricles (11.8 ± 0.6 mm) and the mean lengths of the two principal axes of the largest coronal slices (5.5 ± 0.2 mm and 7.0 ± 0.4 mm), a tri-axial ellipsoid measuring 5.5 × 7 × 12 mm was designed. The prospective location of optical fibers and electrodes on the ellipsoid was defined by a circle-packing algorithm (Rhino CAD; McNeel, Europe) that produced, after cutting away the upper 25% of the ellipsoid, 358 evenly spaced measurement sites at a predefined pitch of 0.7 mm. These locations were assigned to 64 electrical and 294 optical measurements sites that formed repetitive 1-stigma-6-petal patterns. Starting from this general model, the final container exhibiting a wall thickness of 6.5 mm was designed with the inner aspect being expanded such that it was separated by 1.5 mm from the prospective heart surface (Supplementary Fig. 1). Subsequently, orthogonal channels for probe insertion were defined and mounting features permitting fixation of the container to the experimental platform were added (Rhino CAD, McNeel Europe). Finally, the container was split in half and a recess for magnets easing the reproducible assembly of the two halves during experiments was added (Fig. 1d). The container parts were produced using 3D stereolithography (VeroDent, TrigonArt, Germany).

Optical fibers (PMMA, PJR-FB500, N.A. 0.63, Toray, Japan; core diameter: 0.5 mm; diameter with jacket: 1 mm) were cut into ~50 cm long pieces and, following alignment, were fixed in place by a vice. The end of the assembly was face-milled with a diamond router which resulted in orthogonally oriented, optically clear fiber ends. The jacket was stripped from the fibers over a distance of 11 mm.

PTFE-coated silver wires (core diameter: 0.38 mm; diameter with coat: 0.5 mm; AG-15T, Science Products, Hofheim, Germany) were cut into ~10 cm long pieces with the tip facing the heart being ground at an angle of 90° to prevent tissue injury. Care was taken to preserve the PTFE coating up to the tip of the electrode to retain high electrical isolation up to the heart surface. The PTFE coat of the wires was subjected to sodium etching (FluoroEtch^®^ Safety Solvent, Action Technologies, Pennsylvania, USA) to permit glue fixation to the container. Before experiments, electrodes were chlorinated by filling the heart container with 1 mol/L KCL solution and by applying a square-wave current pattern (20 × 2 s pulses of + −5 V, 50% duty cycle) to all electrodes. The average impedance after chlorination amounted to 1.72 ± 1.04 kOhm @ 1 kHz (*n* = 64; Supplementary Fig. 5).

For final assembly, a solid 3D-printed heart model was mounted in the center of the prospective containment with one container half in place (cf. Fig. 1c). Subsequently, the processed optical fibers and electrical wires were inserted into the preformed channels until touching the 3D heart model and then fixed in place by a blackened two-component adhesive (Araldit Standard, Huntsman Cooperation, Texas, USA). Removal of the model exposed the cavity in contact with the hearts during experiments with its surface being formed by closely packed optical fibers and electrodes (cf. image on the right of Fig. 1c). The gap between the cavity surface and the inner container wall (1.5 mm) served the exchange of solution at the heart surface by acting as a drain for the perfusion solution. The assembly of the POEMS system was completed by fixing each heart container half to a fluid reservoir that was connected to the platform holding the printed circuit boards (PCBs) for the electrical connections. Precise coupling of the two platform halves during experiments was eased by alignment pins and magnets (cf. Fig. 1d).

### Electrical subsystem

The silver wires of each container half were soldered to the PCBs that routed the signals to a custom-built electrical recording and stimulation system (cf. Fig.1e). The electrical subsystem was based on four multifunctional integrated circuits (RHS2116, Intan Technologies, USA) that provided 64 analog input/output channels and signal processing functions (filtering and 16-bit A/D conversion at 10 kHz). For the experiments presented, low- and high-pass filters were chosen such as to form a bandpass (100 Hz–5 kHz). The integrated circuits also produced user-defined source- and sink currents (10 nA–2.55 mA) that could be delivered to any of the 64 electrodes for stimulation purposes. Stimulation artifacts were minimized by transiently increasing the AC-coupling frequency of the amplifiers from 0.1 to 1 kHz during stimulation and by manual fine adjustment of the amplitudes of the positive and negative currents of the biphasic stimulation pulse. The custom-developed FPGA design interfaced the signal processing and acquisition units to a PC via an USB3.0 link. A graphical user interface developed in C# served to control the acquisition and stimulation protocols including, e.g., the setting of filter parameters and recording durations as well as the definition of stimulation parameters (amplitudes, shape, channel#, and timing; Supplementary Fig. 6). Moreover, the program supported the visualization of selected signals in real time for continuous monitoring of the functional status of the hearts during ongoing experiments. The recorded data were saved in binary format and included detailed header information regarding the experimental protocol.

### Analysis of electrical data

Offline analysis of unipolar electrograms included the determination of the amplitude and duration of the negative deflection (“downstroke”). Savitzky–Golay filtering of the data returned the maximal slope of the downstroke with the time of its occurrence defining the local activation time (eLAT_SG_). Extracted parameters were mapped onto the ventricular surface of the heart and, for eLAT_SG_ values, activation maps were constructed. Conduction velocities (*θ*) were calculated based on a vector algorithm that determined LATs along a chosen trajectory approximated by the vertices forming the shortest path. For each vertex, the eLAT was determined and plotted as a function of its distance from the origin. The resulting data series was fitted by a line with the slope indicating *θ* (m/s) and the coefficient of determination *r*^2^ being a measure of the uniformity of conduction along the trajectory.

Electrodes that failed to produce electrograms suited for the extraction of LATs due to excess noise or the presence of interfering stimulation artifacts were excluded from the analysis based on visual inspection of the raw data.

### Optical subsystem

The distal ends of the optical fibers were stripped over a distance of 100 mm and assembled into planar sheets containing sequentially ordered fibers that followed coronal sections of the container (cf. Fig. 1e). Each sheet was fixed by a vice and fibers bonded with two-component adhesive (Araldit Standard, Huntsman Cooperation, Texas, USA). The resulting fiber sheets (20) were stacked in a precision-milled vice and bonded using low-viscosity blackened 2-component epoxy (OE121 black, EPO-TEK, USA). The resulting optical light guide combiner (OLG) was face-milled with a diamond router which created an optically clear faceplate that contained all-optical fibers within an area measuring 8  × 9 mm. The OLG was mounted in the focal plane of a custom-made 1× tandem-lens epifluorescence macroscope built around two 50-mm lenses (Rodenstock) that imaged the optical fiber outputs onto the sensor of a fast CMOS camera (sensor area: 10 × 10 mm; frame rates ≤10 kHz; MiCAM Ultima, SciMedia, USA). Excitation light was provided by a 250 W tungsten halogen lamp (XENOPHOT HLX, Osram, Germany). Infrared light was blocked by a hot mirror. Light exposure was controlled by a shutter (SHR; CS65S3S0, Vincent Associates, USA) mounted in front of the excitation filter. Excitation light was deflected towards the fiber-optic array by a dichroic mirror and focused onto the faceplate of the array by the objective. Two filter combinations were used in the experiments: LP495/585/661 ± 81 nm for di-8-ANEPPS experiments and 480 ± 20/505/535 ± 25 for ArcLight-Q239 and ASAP1 experiments (Chroma Technology Corp., Vermont, USA). Optical recordings were controlled by the proprietary MiCAM software and recordings were saved in the respective data format before being sent to the custom POEMS software (POEMSAnalyzer2) for offline analysis.

Synchronization of optical and electrical measurements was realized by mapping the TTL status signal of the MiCAM system to the trigger input of the electrical subsystem and by saving this information together with the electrical data.

### Analysis of optical data

Offline processing and data visualization were accomplished by custom-developed software written in C#. Optical data were automatically assigned to individual fibers by using an extraction mask derived from the image of the fiber-optic faceplate (cf. Fig. 1e). From the 5 × 5 pixels mapping a single optical fiber, the central 3 × 3 pixels were averaged using selectable spatial filters (mean, Gaussian) and the resulting signal assigned to the corresponding fiber. Processing of optical data included drift correction by polynomial fitting, temporal filtering, extraction of basic signal parameters like fractional fluorescence change (Δ*F/F*) per action potential (AP), maximal AP upstroke velocity, and AP duration at defined levels of repolarization. Maximal upstroke velocities of optically recorded APs were obtained from the Savitzky–Golay derivative of the normalized action potential amplitude (APA = 100%) and expressed accordingly as %APA/ms. Under the assumption of an APA of 100 mV, this value translates directly into the customary unit of V/s. The time of the peak of the Savitzky–Golay derivative served as local activation time for optical signals (oLAT_SG_). Alternatively, oLAT was equated with the time when the optical AP upstroke reached 50% of its entire amplitude (oLAT_50%_). Based on these values, panoramic optical activation maps were generated and conduction velocities computed according to the method described above. Signal-to-noise ratios (SNRs) in respect to APs were calculated by dividing the AP amplitude by the standard deviation of a 50 ms recording period obtained at rest. Optical signals were excluded from the analysis in case of saturation.

### Animal models

Validation of the POEMS system was performed with the following four animal models: (1) C57BL/6J wild-type mice (Charles River Laboratories, Sulzfeld, Germany) served as models throughout the development and verification of the POEMS system. (2 and 3) For testing the suitability of the POEMS system to record signals from genetically engineered voltage indicators (GEVIs), Cre-inducible ArcLight-Q239^17^ and ASAP1^16^ transgenic mouse models were developed (cf. below) and crossbred with mice expressing Cre under control of the Myh6 promoter (B6.FVB-Tg(Myh6-cre)2182Mds/J; Charles River Laboratories, Sulzfeld, Germany). This resulted in two transgenic animal models (Myh6-Cre^Tg^/ArcLight-Q239^lox/wt^ and Myh6-Cre^Tg^/ASAP1^lox/wt^) with the respective voltage reporters being expressed in cardiomyocytes. (4) For testing the suitability of the system for optical stimulation of hearts, ReaChR was expressed in cardiomyocytes by crossbreeding mice floxed for ReaChR (B6.Cg-Gt(ROSA)26Sor^tm2.2Ksvo^/J; Charles River Laboratories, Sulzfeld, Germany) with Myh6-Cre mice (Myh6-Cre^Tg^/ReaChR^lox/wt^)^18^. Mice were housed according to Swiss Animal Welfare regulations in an enriched environment with temperature and humidity being controlled between 21–23 °C and 40–60%, respectively, and a light/dark cycle of 12 h/12 h.

All animals were genotyped using protocols developed in-house (cf. below) and as provided by Charles River Laboratories.

### Organ preparation and mounting

Experiments with Langendorff-perfused mouse hearts from animals of either sex were conducted according to federal regulations for animal experimentation under license BE27/17 of the State Veterinary Department of the Canton of Bern, Switzerland. In brief, mice were anesthetized by isoflurane inhalation (Baxter Deutschland GmbH, Unterschleissheim, Germany) followed by intraperitoneal injection of 0.5 mg/kg of 1000 IE/ml heparin (Bichsel AG, Interlaken, Switzerland) to prevent thrombus formation. After 5 min, animals were euthanized by cervical dislocation and, following thoracotomy, hearts were removed and immediately placed in a temperature-controlled (4 °C) titanium dish containing modified Krebs–Henseleit buffer. Following cannulation with a 22-gauge aortic cannula filled with cooled buffer, the hearts were connected to the Langendorff perfusion system and left to equilibrate for 10 min. During this period, preparations underwent gradual warming to 37 °C and, in case of successful cannulation, regular sinus rhythm resumed which, due to altered neurohumoral and loading conditions, exhibited lower frequencies than those present in-vivo^40^. Constant flow (3.5 ml/min) in the Langendorff system was delivered by a peristaltic pump (ISM597D, Ismatec, Wertheim, Germany) while constant temperature (37 °C) was provided by a heat exchanger. The Krebs–Henseleit buffer in the reservoir was continuously bubbled with a gas mixture of 5% CO_2_ and 95% O_2_. A pressure sensor (DPT-100, Utah Medical Products, Utah, USA) connected to a custom-built pressure measurement system was mounted proximal to the cannula and served monitoring of the perfusion pressure during the entire experiments. The perfused hearts were placed into one-half of the POEMS container with the LAD being aligned with the left edge of the container. After adding the second half of the container, continuous electrogram measurements aimed at following the stabilization of the preparations were immediately started. For voltage-sensitive dye (VSD) experiments, the staining solution was added as a bolus (1 ml) to the perfusion solution through a T-joint proximal to the aortic cannula during 15 s. Measurements were started after an equilibration period of 15–20 min. The age of the mice used in the experiments shown ranged from 2 to 9 months (5.6 ± 2.5 months; *n* = 5; 3 f and 2 m). At the end of the experiments, hearts were removed and the container flushed extensively with bi-distilled water. After 10–20 experiments, the inner face of the container was gently cleaned with a moistened optical wipe. While optical fibers needed no further care, electrodes were re-chlorinated when electrogram quality noticeably decreased.

### Solutions

Krebs–Henseleit buffer (K3753-1L, Sigma-Aldrich, Buchs, Switzerland) was supplemented with sodium bicarbonate (S5761−500G, Sigma-Aldrich, Buchs, Switzerland) and calcium chloride (21075, Fluka Chemie GmbH, Buchs, Switzerland). The final composition of the buffer was, in mmol/L: d-glucose 11.1; magnesium sulfate 1.2; potassium phosphate 1.2; potassium chloride 4.7; sodium chloride 118.1; calcium chloride 1.2; sodium bicarbonate 25.0. the pH of the buffer equilibrated with 95%O_2_/5% CO_2_ was 7.2. To suppress motion, the buffer was supplemented with l-blebbistatin (APExBio, Houston, USA) at 15 µmol/L.

Di-8-ANEPPS (Biotium, USA) staining solution was prepared by diluting a 13.5 mmol/L stock solution of the dye dissolved in 25% pluronic F127 (Lutrol F127, BASF Schweiz AG, Basel, Switzerland) and 75% dimethyl sulfoxide (DMSO) (41640-100 mL, Sigma-Aldrich, Buchs, Switzerland) in 1 ml Krebs–Henseleit buffer as to obtain a 135 µmol/L staining solution.

### Electrical stimulation

Hearts were electrically stimulated with biphasic current pulses that were delivered to defined locations on the epicardium by software-based preselection of any combination of two electrodes. The software permitted the selection of stimulation frequency, current pulse widths (0.2–20 ms), current amplitudes (10 nA–2.55 mA), and pulse polarity. In general, hearts were stimulated with 4-ms long biphasic constant-current pulses at double threshold intensity ranging from 30 to 500 µA.

### Optical stimulation

The red-shifted variant of channelrhodopsin (ReaChR), a non-specific cation channel causing membrane depolarization upon illumination, was used to optically stimulate the cardiomyocytes. Excitation light for ReaChR activation was provided through an optical fiber (365 μm, 0.22 NA, FG365LEC, Thorlabs, Newton, USA) coupled to a 590 nm light-emitting diode (LED) (M590F1, Thorlabs, Newton, USA). For locally defined optical stimulation, the OLG was disconnected from the tandem-lens macroscope and the LED-coupled fiber was manually placed in front of any of the 294 fibers. The driver of the LED (DC2100, Thorlabs, Newton, USA) was externally controlled by an analog signal of variable amplitude and duration provided by a stimulator (SD9, Grass Instrument Co, RI, USA). The stimulator itself was triggered by a digital output of the electrical subsystem system thereby enabling synchronization of ReaChR activation and electrogram recording.

### Generation of ArcLight and ASAP1 R26 KI mice

For the generation of ArcLight (B6J;Gt(ROSA)26Sor^tm1(LSL-CLC/ArcLight)Ltk^) and ASAP1 B6J;Gt(ROSA)26Sor^tm1(LSL-ASAP1)Ltk^) knock-in mice, a modified approach targeting the xbaI site in the first intron of the Rosa26 locus was employed^41^.

#### Cloning of targeting vectors

Targeting vectors were cloned by replacing the attR1/2 flanked destination cassette and the IRES-GFP cassette of a Rosa26 targeting vector containing a loxP-flanked STOP cassette and an IRES-GFP reporter (Addgene plasmid 74281^41^) with ASAP^16^, and ArcLight-Q239^17^ coding sequence. Briefly, the destination cassette in the R26 targeting vector (Addgene plasmid 7428) was excised using AscI, followed by a digestion-ligation reaction with KpnI and XmaI in presence of annealed oligos (5′-TCACTTAAGTGCCGGCCGG-3′ and 5′-CCGGCCGGCCGGCACTTAAGTGAGTAC-3′) to remove the IRES-GFP coding sequence. Vectors with ASAP1 and ArcLight-Q239 expression cassettes had been gene-synthesized previously with a commercial supplier (GeneArt, ThermoFisher). ASAP1 was amplified from these vectors using primer 5′-CATACATTATACGAAGTTATCGGCGCGCCACTTTGTACAAAAAAGCAGG-3′ and 5′-GGGAGCTCTCCGGATCCCGGAGGCGCGCCGCTATGTCACGACCTCGAGCT-3′. ArcLight-Q239 was amplified using 5′-CATACATTATACGAAGTTATCGGCGCGCCACTTTGTACAAAAAAGCAGG-3′ and 5′- GGGAGCTCTCCGGATCCCGGAGGCGCGCCGCTACTTATACAGCTCGTCCA-3′. Each PCR product was ligated into the Rosa26 plasmid backbone at the remaining AscI site. Both final targeting vectors Rosa26_ASAP1 and Rosa26_ArcLight-Q239 have been verified by sanger sequencing.

#### Preparation of RNP/HDR donor injection mixes

Lyophilized crRNA and tracrRNA (Alt-R crispr, iDT) were resuspended in 1× microinjection buffer (in mmol/L: TrisHCl 10, EDTA 0.1, pH 7.5) to a final concentration of 10 µmol/L. 1.84 µl of tracr and crRNA were mixed with 10× injection buffer (5 µl) and 0.5 µl of streptococcus pyogenes Cas9 protein (Engen Cas9 NLS, 20 µmol/L, New England Biosciences) and subsequently incubated for 15 min at 37 °C. After incubation, 500 ng of targeting vector were added and the mix was diluted with ddH_2_O to a final volume of 50 µl. To sediment any debris before injection, the final mix was spun at 21,000×*g* for 3 min at room temperature. The injection mix was kept at RT during the microinjection procedure.

#### Microinjection of one-cell embryos

Microinjection approved under license 177-G by the State Veterinary Department of the Canton of Zurich, Switzerland, was performed at the transgenesis core of the University of Zurich, Institute of Laboratory Animal Science. C57BL/6J mice (3–4 weeks old; Charles River, Germany) were superovulated by intraperitoneal injection of 5 IU pregnant mare serum gonadotropin (Folligon, MSD Animal Health GmbH, Luzern, Switzerland) followed 48 h later by injection of 5 IU human chorionic gonadotropin (Pregnyl MSD Animal Health GmbH, Luzern, Switzerland). Mouse zygotes were obtained by mating C57BL/6J stud males with superovulated C57BL/6J females. Zygote microinjections, embryo culture, and retransfer into pseudopregnant foster animals were performed according to standard transgenesis protocols^42^.

#### Characterization of founders

Initial screening of founder animals was performed via PCR using the following primers Arc forward: 5′-GAGTACGTGCAAGAGACA-3′ and Arc reverse: 5′-TAAGCCTGCCCAGAAGACTCC-3′ as well as ASAP1 forward: 5′-TCTGGATCTGACATGGTA-3′ and ASAP1 reverse: 5′-GTTCTGCTGGTAGTGGTCG-3′. Correct integration at the R26 locus was confirmed via southern blot as previously described^41^ and via PCR over the 5′-homology arm with primers 5′-R26 forward: 5′-AAGACCGCGAAGAGTTTGTCC-3′ and 5′-R26 forward: 5′-TCAGACAGCAGAAATATAGCC-3′.

#### Maintenance

For regular breeding purposes, animals were genotyped using the following primers: ArcLight-Q239: Arc forward: 5′-GAGTACGTGCAAGAGACA-3′ and Arc rev: 5′-TAAGCCTGCCCAGAAGACTCC-3′ tg: 1271 bp amplicon. ASAP1: ASAP1 fwd: 5′-TCTGGATCTGACATGGTA-3′ and ASAP1 rev: 5′-GTTCTGCTGGTAGTGGTCG-3′ tg: 667 bp amplicon. Founders were backcrossed to C57BL/6J for two generations before phenotypic analysis.

A list of all primers used for the generation of transgenic animals and for routine genotyping is provided in Supplementary Table 1.

### Statistical analyses

Data are given as mean ± standard deviation with “*n*” referring to the number of signals acquired simultaneously by electrical and optical recording sites during a single activation of the heart under investigation. Normal distribution of data was assessed using the Shapiro–Wilk test. Data comparison was performed with the Student’s *t* test (homoscedastic or heteroscedastic where appropriate). Differences were considered significant at *P* < 0.05. Statistical analyses were conducted with Excel (Microsoft) and MinitabExpress (Minitab LLC). Graphs were constructed with Graphpad Prism (Graphpad Software LLC) and figures compiled with Canvas X Draw (Canvas GFX, Inc.).

### Ethics declaration

Experiments with animals were conducted in accordance with relevant ethical regulations for animal testing and research as required by federal regulations. The study received ethical approval by the committee on animal experimentation of the State Veterinary Department of the Canton of Bern, Switzerland (license BE27/17) and of the State Veterinary Department of the Canton of Zurich, Switzerland (license 177-G).

### Reporting summary

Further information on research design is available in the Nature Research Reporting Summary linked to this article.

## Supplementary information

Supplementary Information

Reporting Summary

Supplementary Movie 1

Supplementary Movie 2

Supplementary Movie 3

Supplementary Movie 4

Description of Additional Supplementary Files

## Data Availability

The data generated in this study, 3D print files of the heart container, the PCB layout of the electrode connection pad, and a step-by-step protocol have been deposited and can be accessed under 10.5281/zenodo.5501068. Source data are provided with this paper.

## Source data

Source Data

## Code availability

The software code for the analysis of data generated by the POEMS system (POEMSAnalyzer2) can be accessed under 10.5281/zenodo.5501068. Source data are provided with this paper.
