# Supplementary material for: Enabling comprehensive optogenetic studies of mouse hearts by simultaneous opto-electrical panoramic mapping and stimulation
Source: Nat Commun. 2021 Oct 4;12:5804. doi: 10.1038/s41467-021-26039-8 (PMC8490461; doi:10.1038/s41467-021-26039-8)
Supplement: Supplementary file 1 — Supplementary Information [file 41467_2021_26039_MOESM1_ESM.pdf]

Supplementary Information

Enabling comprehensive optogenetic studies of mouse hearts by simultaneous opto-electrical panoramic mapping and stimulation

Michael Rieger[1], Christian Dellenbach[1], Johannes vom Berg[2], Jane Beil-Wagner[2], Ange Maguy[1], Stephan Rohr[1]*

[1]Department of Physiology, University of Bern, Bühlplatz 5, Bern, Switzerland

[2]Institute of Laboratory Animal Science, University of Zurich, Wagistrasse 12, Schlieren, Switzerland

*Correspondence

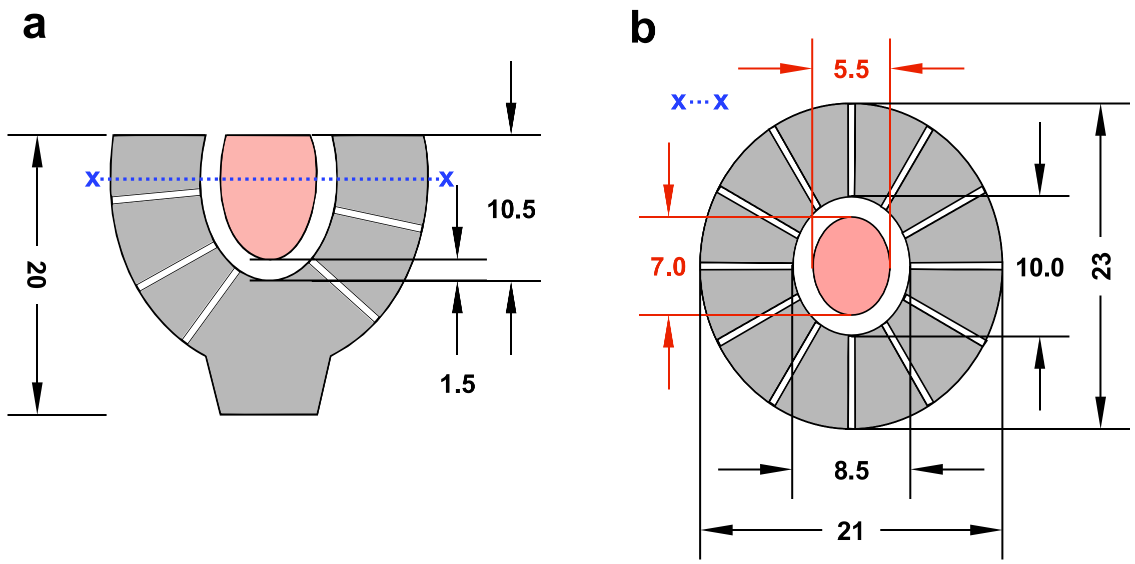

Supplementary Fig. 1

Dimensioning of the heart container. Positioning of the heart (red) in respect to the heart container (grey; dimensions in mm). **a** Longitudinal section. **b** Cross-section at the level of the stippled blue line depicted in (a).

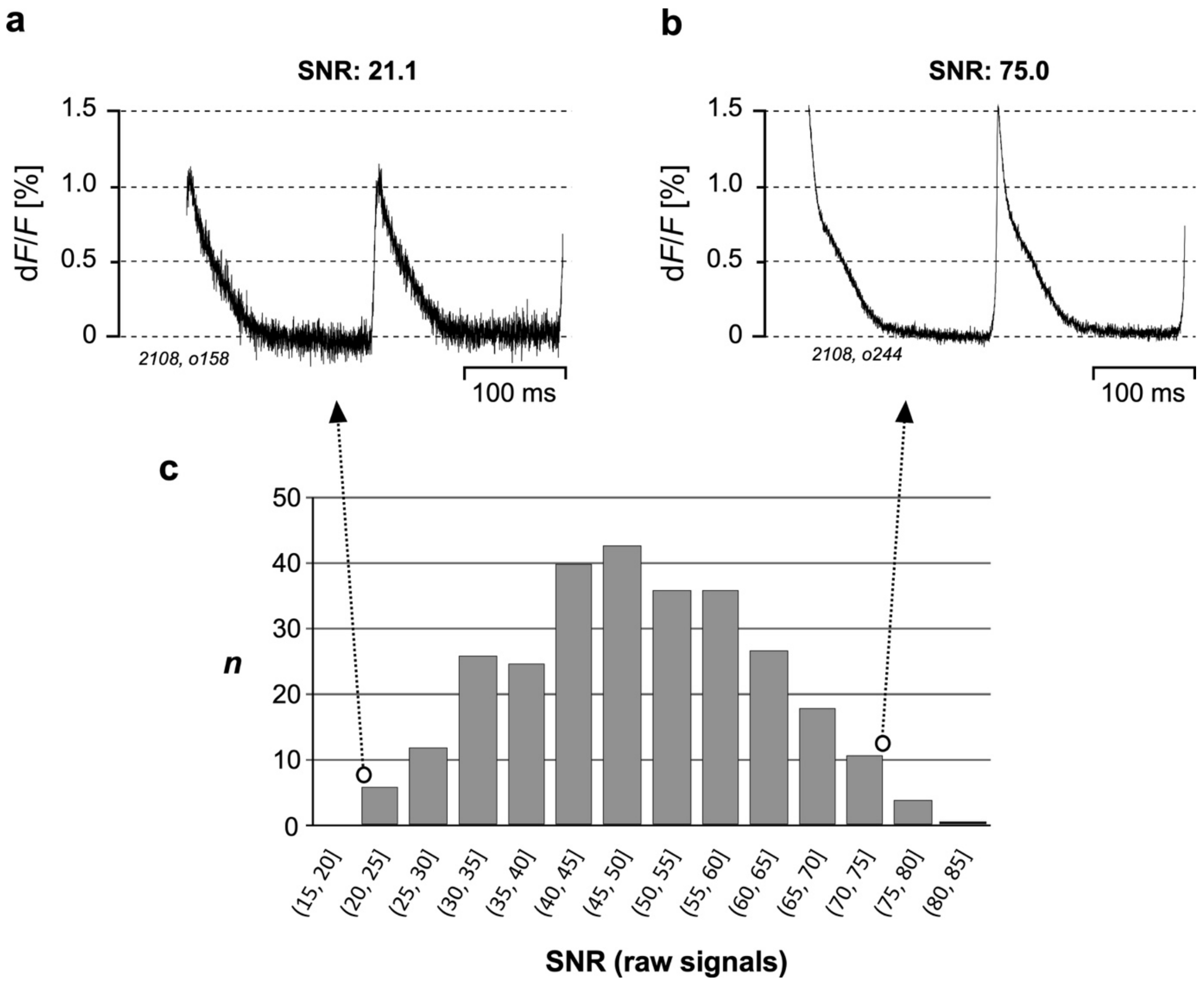

Supplementary Fig. 2

Signal-to-noise ratios of single shot action potential recordings from a di-8-ANEPPS stained heart. Examples of oAPs exhibiting **a,** modest and **b,** high signal-to-noise ratios (SNR). **c,** Distribution of SNRs of all optical fibers in response to a single ventricular activation (framerate: 10 kHz). Source data are provided as a Source Data file.

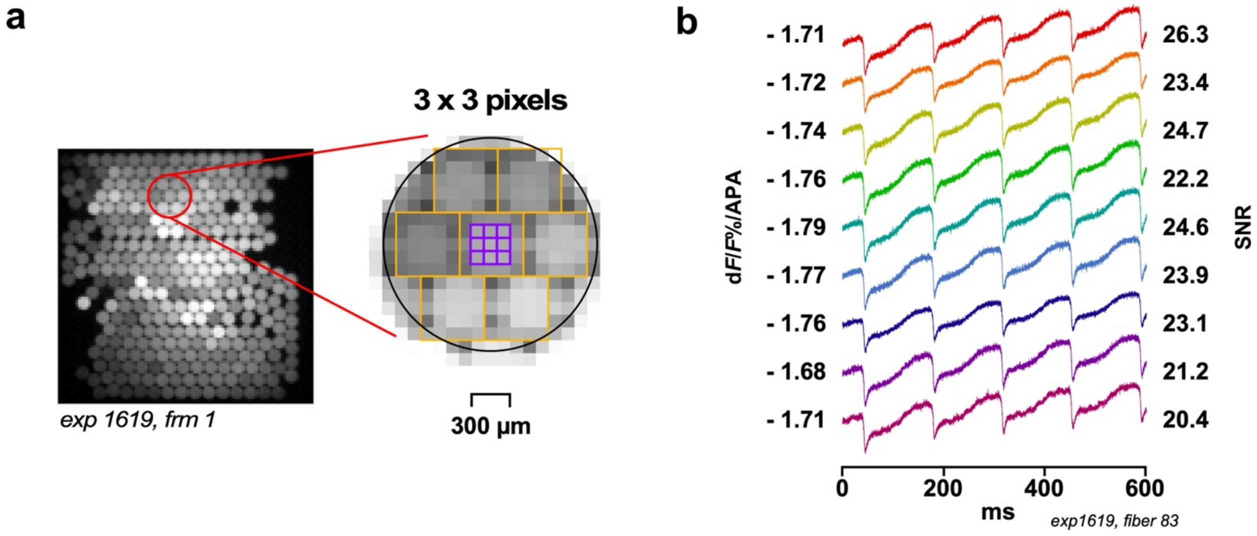

Supplementary Fig. 3

Single pixel signals from a di-8-ANEPPS stained heart. a, Fiber array faceplate with magnification showing the central fiber together with the position of the 9 pixels receiving input from this fiber. **b,** Sequence of action potentials as recorded by the 9 pixels. The raw signals shown were acquired at 10 kHz (d*F*/*F*%/APA: fractional fluorescence change per action potential amplitude; SNR: signal-to-noise ratio).

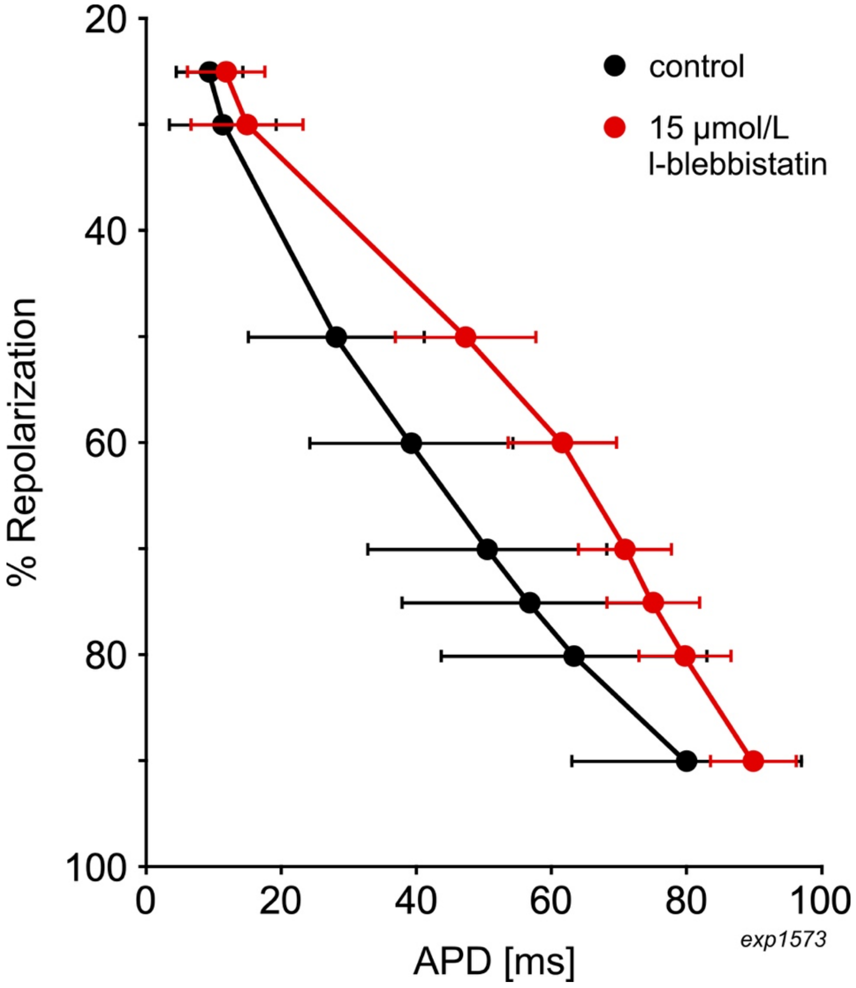

Supplementary Fig. 4

APD prolongation by l-blebbistatin. Action potential duration (APD) measured at indicated levels of repolarization were prolonged in presence of l-blebbistatin (average ± SD, *n*=261; paired data are significantly different at $p < 1.4 \cdot 10^{-6}$; *n*=261). Source data are provided as a Source Data file.

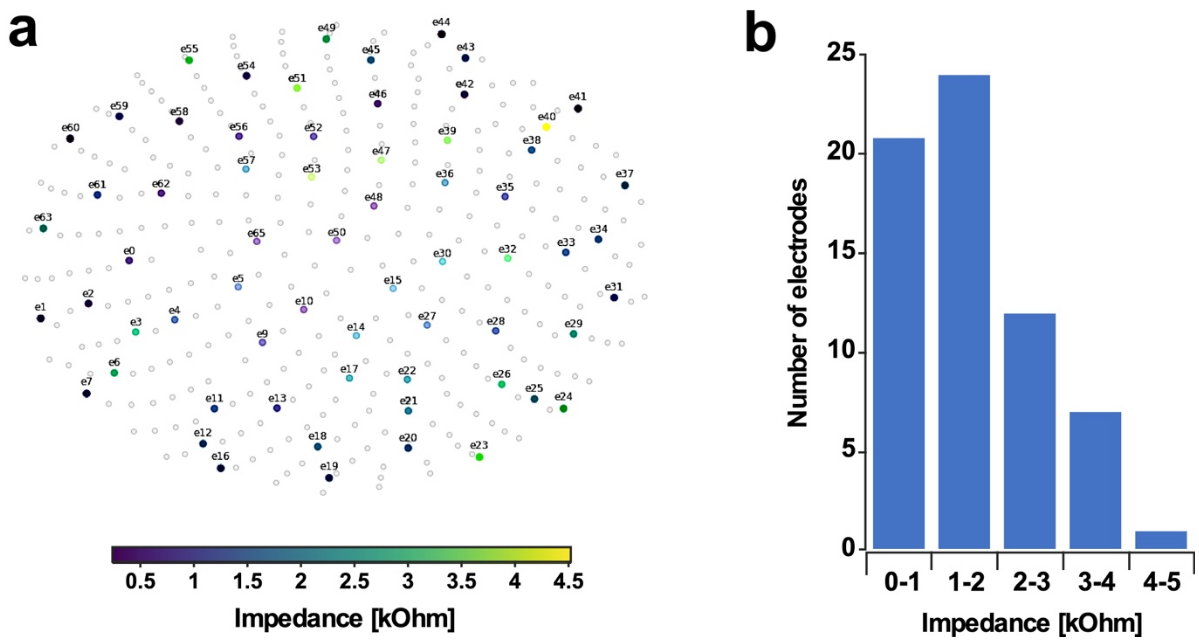

Supplementary Fig. 5

Electrode impedance. a Planar projection of the electrode layout with color coded impedance values. **b** Histogram of impedances (1.72 ± 1.05 kΩ; mean ± SD; *n*=64). Impedances were measured by applying a 1 kHz sinusoidal signal to the electrodes of the heart container filled with Hank's balanced salt solution. Source data are provided as a Source Data file.

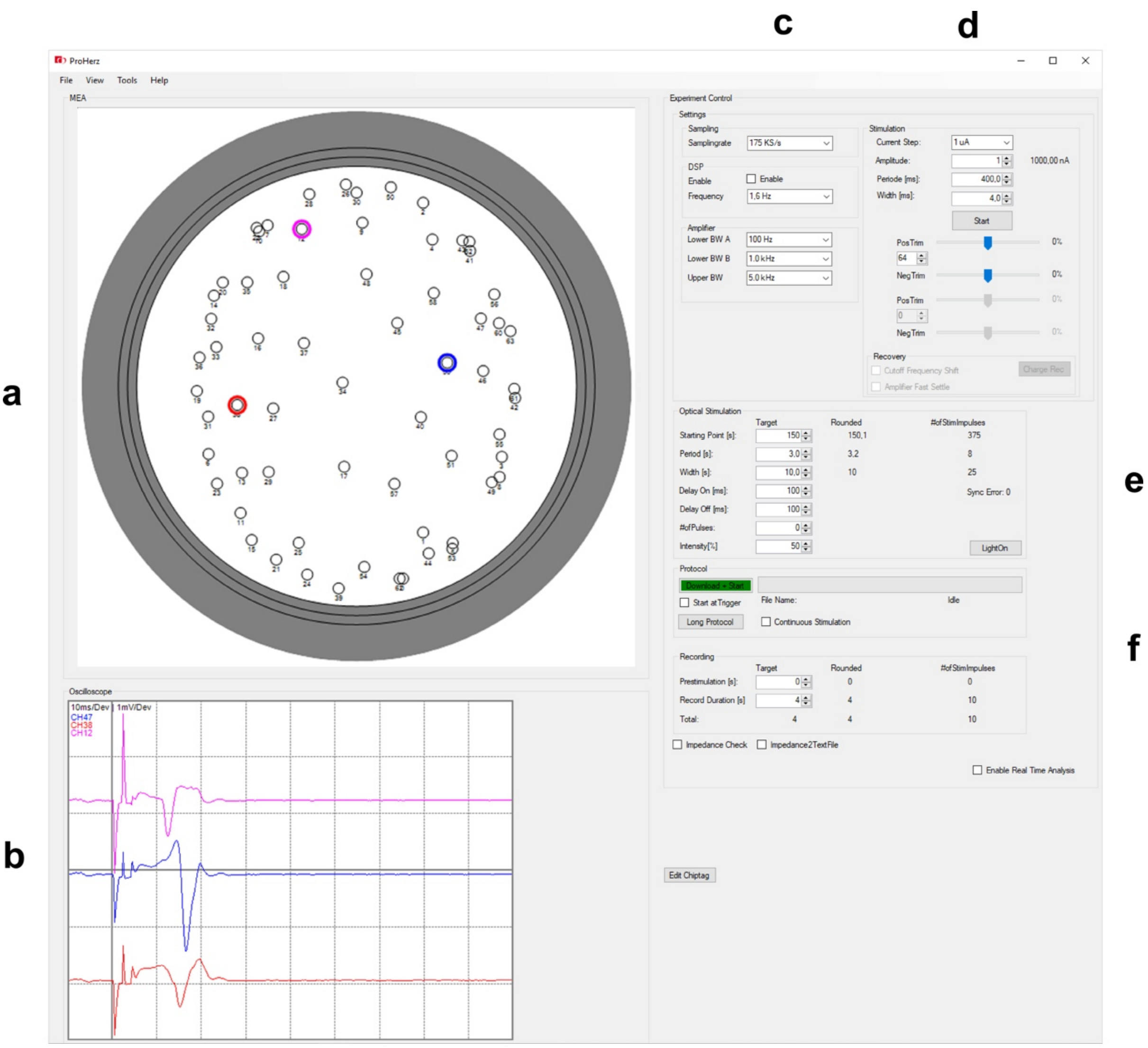

Supplementary Fig. 6

Electrical subsystem control. a Planar projection of the electrode layout that permits the selection of individual electrodes for real-time data display and stimulation. **b** Real-time display of signals recorded by the electrodes selected in (a). **c-f** Control panels for electrical data acquisition, electrical stimulation, optical stimulation and data management including selection of single or synchronized recordings and start of experiments.

Name	Purpose	5`-->3` Sequence
	**Generation of ArcLight and ASAP1 R26 KI mice**	
GFPrem fwd	Removing IRES GFP from R26 Addgene Nr 7428	TCACTTAAGTGCCGGCCGG
GFPrem rev		CCGGCCGGCCGGCACTTAAGTGAGTAC
ASAP1_Arcout fwd	Amplyfing ASAP1 expression cassette from GeneArt vector	CATACATTATACGAAGTTATCGGCGCGCCACTTTGTACAAAAAAGCAGG
ASAP1out rev		GGGAGCTCTCCGGATCCCGGAGGCGCGCCGCTATGTCACGACCTCGAGCT
ASAP1_Arcout fwd	Amplyfing ArcLight expression cassette from GeneArt vector	CATACATTATACGAAGTTATCGGCGCGCCACTTTGTACAAAAAAGCAGG
Arcout rev		GGGAGCTCTCCGGATCCCGGAGGCGCGCCGCTACTTATACAGCTCGTCCA
5'R26 fwd	R26 5`homology arm	AAGACCGCGAAGAGTTTGTCC
5'R26 rev		TCAGACAGCAGAAATATAGCC
Arc fwd	Founder characterization ArcLight R26 KI allele	GAGTACGTGCAAGAGACA
Arc rev		TAAGCCTGCCCAGAAGACTCC
ASAP1 fwd	Founder characterization ASAP1 R26 KI allele	TCTGGATCTGACATGGTA
ASAP1 rev		GTTCTGCTGGTAGTGGTCG
	**Genotyping / Crossbreeding**	
Arc fwd	Genotyping ArcLight KI allele	GAGTACGTGCAAGAGACA
Arc rev		TAAGCCTGCCCAGAAGACTCC
ASAP1 fwd	Genotyping ASAP1 KI allele	TCTGGATCTGACATGGTA
ASAP1 rev		GTTCTGCTGGTAGTGGTCG
Myh6-Cre fwd	Genotpying M yh6-Cre transgene	ATGACAGACAGATCCCTCCTATCTCC
Myh6-Cre rev		CTCATCACTCGTTGCATCATCGAC
ReaChR fwd	Genotpying ReaChR KI allele	CTTCCCTCGTGATCTGCAAC
ReaChR rev		GTTATGTAACGCGGAACTCCA

Supplementary Table 1

List of primers used for the generation of the transgenic animals and for genotyping**.**
