# Supplementary material for: Enabling comprehensive optogenetic studies of mouse hearts by simultaneous opto-electrical panoramic mapping and stimulation
Source: Nat Commun. 2021 Oct 4;12:5804. doi: 10.1038/s41467-021-26039-8 (PMC8490461; doi:10.1038/s41467-021-26039-8)
Supplement: Supplementary file 2 — Reporting Summary [file 41467_2021_26039_MOESM2_ESM.pdf]

nature portfolio

Corresponding author(s): Stephan Rohr

Last updated by author(s): Sep 10, 2021

Reporting Summary

Nature Portfolio wishes to improve the reproducibility of the work that we publish. This form provides structure for consistency and transparency in reporting. For further information on Nature Portfolio policies, see our Editorial Policies and the Editorial Policy Checklist.

Statistics

For all statistical analyses, confirm that the following items are present in the figure legend, table legend, main text, or Methods section.

n/a	Confirmed	
☐	☒	The exact sample size (*n*) for each experimental group/condition, given as a discrete number and unit of measurement
☐	☒	A statement on whether measurements were taken from distinct samples or whether the same sample was measured repeatedly
☐	☒	The statistical test(s) used AND whether they are one- or two-sided<br>*Only common tests should be described solely by name; describe more complex techniques in the Methods section.*
☒	☐	A description of all covariates tested
☐	☒	A description of any assumptions or corrections, such as tests of normality and adjustment for multiple comparisons
☐	☒	A full description of the statistical parameters including central tendency (e.g. means) or other basic estimates (e.g. regression coefficient) AND variation (e.g. standard deviation) or associated estimates of uncertainty (e.g. confidence intervals)
☐	☒	For null hypothesis testing, the test statistic (e.g. *F*, *t*, *r*) with confidence intervals, effect sizes, degrees of freedom and *P* value noted<br>*Give P values as exact values whenever suitable.*
☒	☐	For Bayesian analysis, information on the choice of priors and Markov chain Monte Carlo settings
☒	☐	For hierarchical and complex designs, identification of the appropriate level for tests and full reporting of outcomes
☒	☐	Estimates of effect sizes (e.g. Cohen's *d*, Pearson's *r*), indicating how they were calculated

Our web collection on statistics for biologists contains articles on many of the points above.

Software and code

Policy information about availability of computer code

Data collection	Optical data: Proprietary software of the camera manufacturer ('micam_ultima_usb1', V1204).<br>Electrical data: Proprietary software written for our custom-developed hardware (64 channel multi-electrode array recording system).<br>Extraction of relevant parameters from the data generated by the optical and electrical data acquisition systems was handled by the custom-developed analysis software, 'POEMSAnalyzer2'. The software code has been deposited and can be accessed at https://doi.org/10.5281/zenodo.5501068. Use of the POEMSAnalyzer2 software requires matching of the input data to the data formats of the camera and multielectrode array recording systems used when replicating the POEMS system.
Data analysis	In general, data analysis was based on well established algorithms being embedded in the 'POEMSAnalyzer2' software.<br>For optimized fiber/electrode placement on the heart container, a circle packaging algorithm was used (built-in function of Rhino CAD, McNeel, Europe, V6.0).<br>The vector algorithm calculates conduction velocities by determining local activation times (LATs) along a chosen trajectory approximated by the vertices forming the shortest path. For each vertex, the LAT is determined and plotted as a function of its distance from the origin. The resulting data series is fitted by a line with the slope indicating the conduction velocity (m/s) with the coefficient of determination r2 being a measure of the uniformity of conduction along the trajectory.<br>The meta-analysis of extracted optical and electrical parameters was performed with Excel for Mac (Microsoft, V16.52) and MinitabExpress (Minitab LLC, V1.5.3). Comparative data analysis was performed with Minitab Express (Minitab LLC, V1.5.3) and Graphpad Prism (GraphPad Software, LLC, V9.1.2). Figures were compiled with Canvas X Draw (Canvas GFX, Inc., V7.0.1).

For manuscripts utilizing custom algorithms or software that are central to the research but not yet described in published literature, software must be made available to editors and reviewers. We strongly encourage code deposition in a community repository (e.g. GitHub). See the Nature Portfolio guidelines for submitting code & software for further information.

Data

Policy information about availability of data

All manuscripts must include a data availability statement. This statement should provide the following information, where applicable:

- Accession codes, unique identifiers, or web links for publicly available datasets
- A description of any restrictions on data availability
- For clinical datasets or third party data, please ensure that the statement adheres to our policy

Data availability statement:
Source data are provided with this paper. The data generated in this study, 3D print files of the heart container, the PCB layout of the electrode connection pad, and a step-by-step protocol have been deposited and can be accessed under https://doi.org/10.5281/zenodo.5501068.

Field-specific reporting

Please select the one below that is the best fit for your research. If you are not sure, read the appropriate sections before making your selection.

- [x] Life sciences
- [ ] Behavioural & social sciences
- [ ] Ecological, evolutionary & environmental sciences

For a reference copy of the document with all sections, see nature.com/documents/nr-reporting-summary-flat.pdf

Life sciences study design

All studies must disclose on these points even when the disclosure is negative.

Sample size	The study describes new hardware permitting simultaneous optical and electrical panoramic mapping and stimulation of mouse hearts. The system was validated with 18 hearts total of which 4 examples are shown in the figures of the study (3 with optical voltage indicators, 1 expressing an optical voltage actuator). Multiple experiments were conducted on each heart (mapping during sinus rhythm and mapping following epicardial electrical or optical stimulation). The sample size for the validation experiments was considered adequate after obtaining positive proof of the functionality of the system with ≥ 3 hearts each for di-8-ANEPPS experiments and the 3 transgene models. The finding that all of these experiments produced consistent and qualitatively identical results with regards to the performance of the optical and electrical subsystem of the POEMS setup fully validates the system.
Data exclusions	Data exclusion criteria are defined in the manuscript (electrical: electrodes with insufficient signal-to-noise ratios; optical: saturated signals).
Replication	Experiments per condition (di-8-ANEPSS, ArcLight Q239, ASAP1 and ReaChR) were replicated with ≥ 3 hearts each.
Randomization	The type of experiments performed (validation of a hardware system) did not require establishment of different experimental groups.
Blinding	The type of experiments performed (validation of a hardware system) did not require blinding of experimenters.

Reporting for specific materials, systems and methods

We require information from authors about some types of materials, experimental systems and methods used in many studies. Here, indicate whether each material, system or method listed is relevant to your study. If you are not sure if a list item applies to your research, read the appropriate section before selecting a response.

Materials & experimental systems

n/a	Involved in the study	
x		Antibodies
x		Eukaryotic cell lines
x		Palaeontology and archaeology
x		Animals and other organisms
x		Human research participants
x		Clinical data
x		Dual use research of concern

Methods

n/a	Involved in the study	
x		ChIP-seq
x		Flow cytometry
x		MRI-based neuroimaging
