# Supplementary material for: Enabling comprehensive optogenetic studies of mouse hearts by simultaneous opto-electrical panoramic mapping and stimulation
Source: Nat Commun. 2021 Oct 4;12:5804. doi: 10.1038/s41467-021-26039-8 (PMC8490461; doi:10.1038/s41467-021-26039-8)
Supplement: Supplementary file 7 — Description of Additional Supplementary Files [file 41467_2021_26039_MOESM7_ESM.pdf]

Title: Supplementary Movie 1 Simultaneous optical and electrical determination of the spread of excitation in a di-8-ANEPPS stained C57BL/6J mouse heart in sinus rhythm.

Description: Upper left: Examples of an optical and an electrical signal recorded in parallel from the heart in sinus rhythm (~ 5 Hz). **Upper right:** Clock. **Lower left:** Animations of the optically and electrically determined spread of the activation wavefront as seen from different aspects of the heart (orientation indicated by the heart model on top: A: azimuth; E: elevation; red: left ventricle; blue: right ventricle). The red and blue dot indicate the positions of the optical and electrical recording sites producing the signals shown in the top left panel. **Lower right:** Animated stereo- and mercator projections of ventricular activation.

Title: Supplementary Movie 2
Description: Excerpt of Supplementary Movie 1 showing a single activation in ultra-slow motion.

Title: Supplementary Movie 3 Electrically stimulated activation sequences of hearts from transgenic mouse models expressing the genetically engineered voltage indicators ArcLight-Q239 and ASAP1 in cardiomyocytes.

Description: Left: Example of a single stimulated activation as recorded from an ArcLight-Q239 expressing heart with optical and electrical traces (top), panoramic views of activation (center) and animated stereo- and mercator projections (bottom). The resting state is shown in blue, the peak of the action potential in red. The blue and red spheres indicate the positions of the stimulation electrodes. The green sphere refers to the recording site of the optical signal shown on top. **Right:** Same for a heart expressing the optogenetic voltage indicator ASAP1 in cardiomyocytes.

Title: Supplementary Movie 4 Optical stimulation of a heart from a transgenic mouse model expressing the depolarizing optogenetic voltage actuator ReaChR in cardiomyocytes.

Description: Left: Control recordings of the heart in sinus rhythm with an example electrogram (top; atrial activation marked with red box), panoramic views of the spread of the activation wavefront (center; green sphere indicates recording site of the signal shown above) and animated stereo- and mercator projections of the spread of the activation wavefront (bottom). **Right:** Same for optically stimulated cardiac activation. The yellow arrows in the top display indicate the time of optical stimulation, the grey disc in the displays below refer to the location of the single fiber optical stimulation (switches transiently to yellow during stimulation).
